# Atrial Fibrillation and Depression: A Bibliometric Analysis From 2001 to 2021

**DOI:** 10.3389/fcvm.2022.775329

**Published:** 2022-02-16

**Authors:** Yuzhen Ai, Yaxuan Xing, Longmei Yan, Dan Ma, Anran Gao, Qiwu Xu, Shan Zhang, Ting Mao, Qiu Pan, Xiaojuan Ma, Jingchun Zhang

**Affiliations:** ^1^Xiyuan Hospital, China Academy of Chinese Medical Sciences, Beijing, China; ^2^National Clinical Research Center for Chinese Medicine Cardiology, Xiyuan Hospital, China Academy of Chinese Medical Sciences, Beijing, China; ^3^Graduate School, Beijing University of Chinese Medicine, Beijing, China

**Keywords:** atrial fibrillation, depression, bibliometric analysis, visual analysis, CiteSpace

## Abstract

**Background:**

The control of diseases related to atrial fibrillation (AF) may reduce the occurrence of AF, delay progression, and reduce complications, which is beneficial to the prevention and treatment of AF. An increasing number of studies have shown that AF is associated with depression. However, to date, there has not been a bibliometric analysis to examine this field systematically. Our study aimed to visualize the publications to determine the hotspots and frontiers in research on AF and depression and provide guidance and reference for further study.

**Methods:**

Publications about AF and depression between 2001 and 2021 were retrieved from the Web of Science Core Collection (WOSCC) database. CiteSpace 5.8. R1, VOSviewer 1.6.16, and Excel 2019 software tools were used to conduct this bibliometric study.

**Results:**

In total, 159 articles and reviews were analyzed. The number of publications has been increased sharply since 2018. David D. McManus had the largest number of publications. The most prolific country was the USA with 54 publications but the centrality was <0.1. The most prolific institution was Northeastern University. Three clusters were formed based on keywords: The first cluster was composed of atrial fibrillation, depression, anxiety, symptoms, ablation, and quality of life, et al. The second cluster were risk, prevalence, mortality, heart failure, association, et al. While the third cluster included anticoagulation, impact, stroke, management, warfarin, et al. After 2019, stroke and prediction are the keywords with strongest citation bursts.

**Conclusion:**

Research on AF and depression is in its infancy. Cooperation and exchanges between countries and institutions must be strengthened in the future. The effect of depression on prevalence and mortality in AF, depression on ablation in AF, and impact of depression on anticoagulation treatment in AF have been the focus of current research. Stroke prevention (including anticoagulant therapy) is the research frontier, which may still be the focus of research in the future.

## Introduction

Atrial fibrillation (AF) is the most common sustained cardiac arrhythmia in clinic, affecting 2–4% of adults (1). Its common clinical manifestations are palpitation, chest tightness, reduced exercise tolerance, etc., that seriously affect the quality of life and emotional status of patients (2). AF is also associated with an increased risk of stroke, heart failure, dementia, and other complications, which poses a significant burden to healthcare systems globally (1). It is still a clinical challenge associated with a high relapse rate and poor prognosis.

Depression is a common psychological comorbidity in patients with cardiovascular disease (CVD), and AF tends to link with higher risks of depressive symptoms rather than coronary heart disease or hypertension (3). The prevalence of depression in patients with AF has been reported as high as 38 to 42.7% (4, 5), which is similar to that in oncology populations (6–8). A number of studies have indicated that depression was closely related to the occurrence and development of AF, which increase the complexity of management and the risk of adverse outcomes in patients with AF (9–11). The AHA scientific statement *Psychological Health, Well-Being, and the Mind-Heart-Body Connection: A Scientific Statement* has clearly stated that interventions to improve psychological health can have a beneficial impact on cardiovascular health (12). The prevention and treatment of comorbidity of AF and depression may reduce the onset of AF, delay its progression, reduce complications, and provide new ideas for its prevention and treatment. However, compared with depression on coronary heart disease, heart failure, ventricular arrhythmia, and cancer, the basic and clinical research on AF and depression is not thorough enough. There is no ideal treatment for comorbidity of AF and depression. The combination of antiarrhythmic drugs and antidepressant drugs has many side effects and poor efficacy (13, 14). In general, in-depth study of the role of depression in AF will help to understand the development and prognosis of AF, and provide new ideas for clinical prevention and treatment. With the development of psycho-cardiology, increasing attention has been paid to the relationship between depression and AF, and studies on this topic have been published continuously. Understanding the current hot spots and frontiers in the research field related to AF and depression is helpful for us to conduct in-depth research.

CiteSpace and VOSviewer are bibliometric analysis software widely used, which can intuitively reveal the dynamic development law of scientific knowledge, help researchers quickly obtain applicable scientific information, and provide practical and valuable references or guidance (15–17). In addition to helping to describe and predict the current situation, hotspots and trends in a specific research field through quantitative analysis, bibliometrics can also identify the productivity of countries, institutions, authors and the cooperative relationship between them, providing researchers with potentially valuable information. Nevertheless, there are few bibliometric studies on AF and depression. We mapped the knowledge map of AF and depression based on bibliometric analysis, aiming to provide ideas for researchers to discover new themes and directions.

## Methods

### Search Strategies

Data were downloaded from the Science Citation Index Expanded database of the Web of Science Core Collection (WoSCC) within 1 day on September 01, 2021. The search formula was set to [TS = (“atrial fibrillation^*^” OR “auricular fibrillation^*^”) AND TS = (“depression^*^” OR “depressive symptom^*^”)], language (English), time horizon: 2001–2021, literature type (Article or Review). Two reviewers (Yuzhen Ai and Yaxuan Xing) independently screened the publications by reading the titles and abstracts in order to exclude the article of which the theme was not related to AF or depression. If a disagreement occurred between them, it was resolved by further reading the full text and consulting with a third investigator (Jingchun Zhang).

### Data Collection and Analysis

The “Full Record and Cited References” of these records were extracted as the format of “Plain Text” into CiteSpace 5.8.R1 software. No duplicate records were identified using the native function of checking duplicate of the software. Keywords were merged with the same meaning in the exported data, such as “risk” and “risk factor” into “risk,” “ablation” and “catheter ablation” into “ablation.” And Meaningless keywords were removed, such as “fibrillation,” “disease,” etc. Microsoft Office Excel 2019 was used to manage data and create the chart of annual research output. CiteSpace 5.8.R1 software was used to carry out bibliometric and visual knowledge map analysis in terms of authors, countries and institutions, co-cited author, co-cited references, and keywords burst detection. The parameters of CiteSpace 5.8.R1 were as follows: time span (2001–2021), years per slice (2), selection criteria (Top 50), Pruning: Pathfinder, Pruning sliced networks, Pruning the merged network. In addition, VOSviewer 1.6.16 software was used to conduct the network analysis of the frequent keywords. The parameters of VOSviewer 1.6.16 were as follows: the minimum number of occurrences of a keyword was 10.

## Results

### General Information and Annual Publication Output

**Figure 1:**
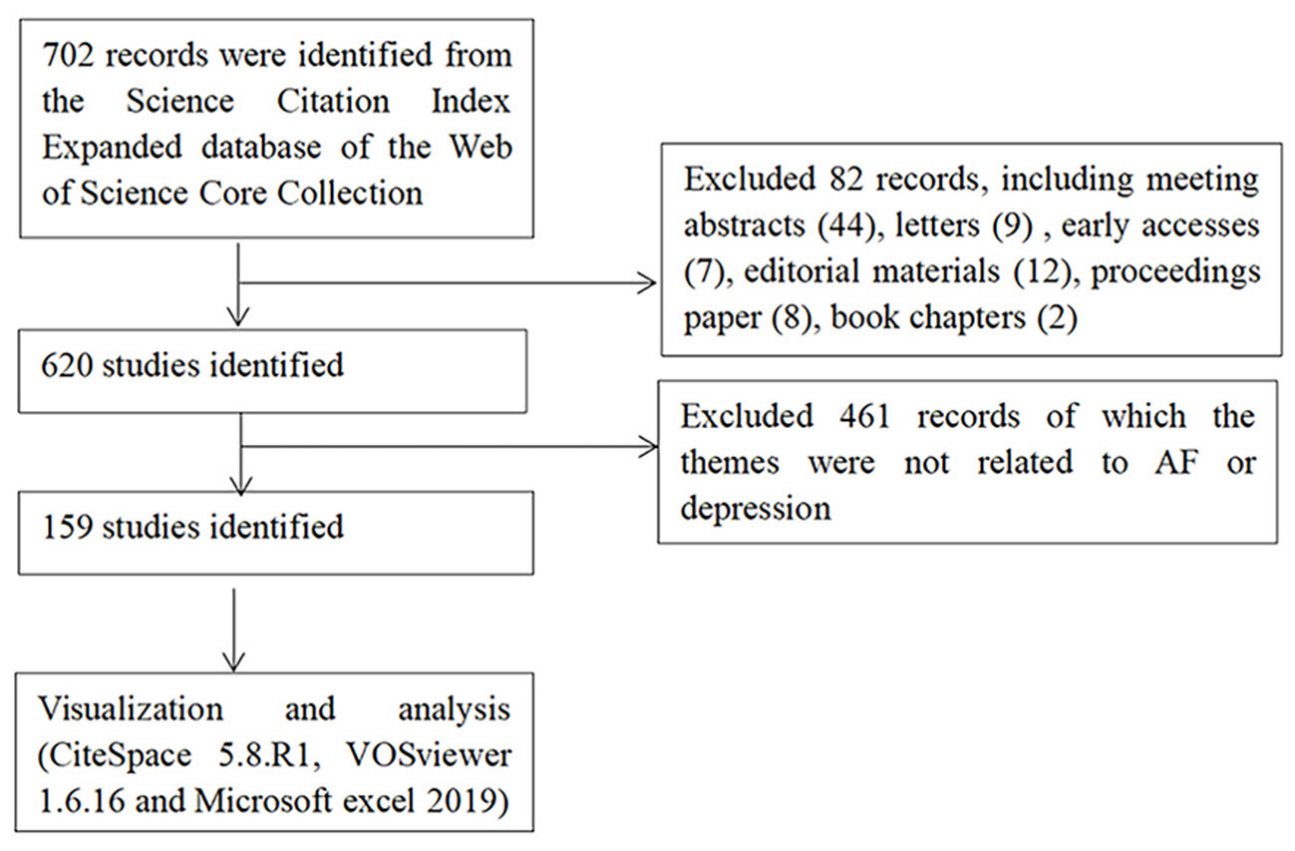
Flowchart of literature selection.

As a result, a total of 702 records were identified from the Science Citation Index Expanded database of WoSCC, and there were 532 articles (75.8%) and 88 reviews (12.5%) among them. We eliminated invalid documents (461) of which the themes were not related to AF or depression. Finally, a total of 159 records including 146 articles (91.8%) and 13 (8.2%) reviews were used as the dataset in this study. Flowchart of literature selection was shown in Figure 1.

**Figure 2:**
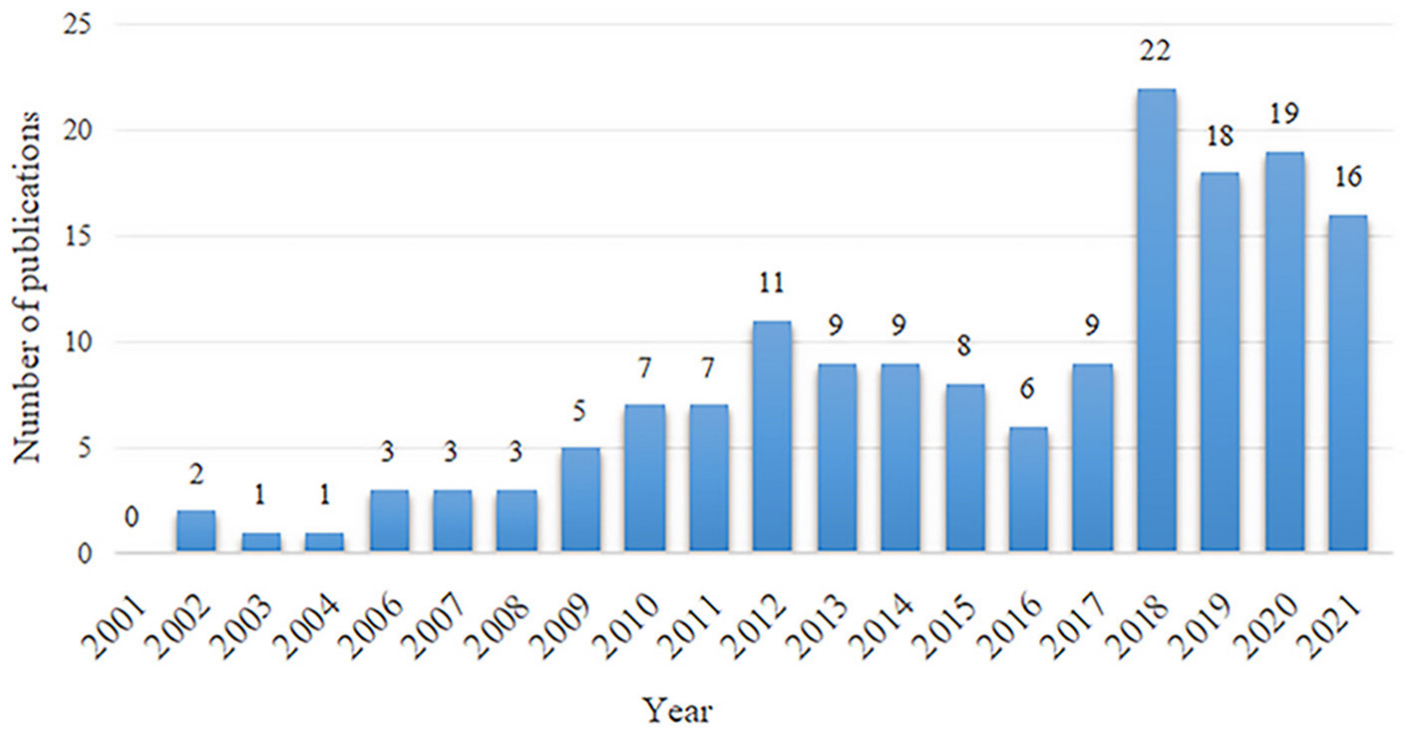
The number of annual publications relating to research about the atrial fibrillation and depression from 2001 to 2021.

We presented the number of annual publications in the past 20 years in the form of a histogram (Figure 2), which reflected the development trend of research in this field. There were not even publications related to AF and depression in 2001. From 2002 to 2012, the number of publications on AF and depression began to increase, with a maximum of 11 publications per year. From 2013 to 2017, the number of annual publications fluctuated between 6 and 9. But, it had been increasing sharply since 2018.

### Distribution of Authors

**Figure 3:**
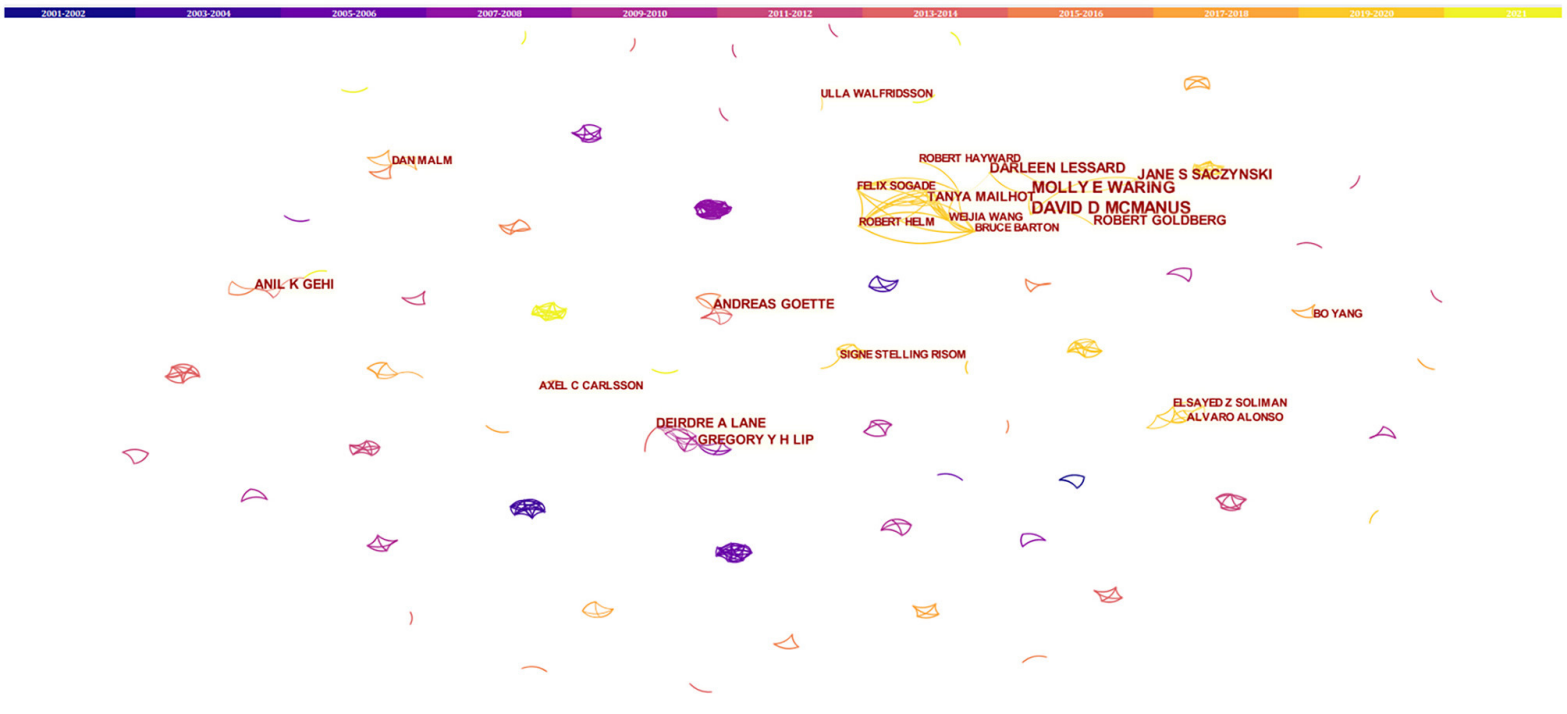
The network of authors contributed to research about atrial fibrillation and depression. In the network, the larger the node was, the more contribution the author had made to that field. The color of the line represents the time of first co-occurrence. The thicker the line is, the greater the connection strength is (calculation method based on cosine).

The top 10 authors published more than or equal to 4 papers on AF and depression, and 6 of them published the related first article in 2019 (Table 1). Interestingly, 7 of the top 10 authors work in the United States. David D. McManus had the largest number of publications (7 publications), and cooperated more with Molly E. Waring, Robert Goldberg, and Darleen Lessard. Centrality is a parameter used to measure the importance of nodes in the network. The higher the centrality of nodes is, the more frequency it contact with other nodes and the more important it is in the whole network. In the network formed by CiteSpace analysis, nodes with centrality >0.1 are called key nodes, and nodes with higher centrality (>0.1) are usually highlighted with purple rings. The lines between the nodes reflect the connections between authors. According to the network of authors contributed to research about AF and depression (Figure 3), there were lots of small teams studying the association between AF and depression, but there are few contacts between teams, and the centrality of each author was much <0.01.

**Table 1:** Top 10 authors on atrial fibrillation and depression.

**Rank**	**Author**	**Count (%)**	**Year**	**Centrality**
1	David D. McManus	7	2019	0.00
2	Molly E. Waling	6	2019	0.00
3	Jane S. Saczynski	5	2019	0.00
4	Darleen Lessard	5	2019	0.00
5	Anil K. Gehi	4	2012	0.00
6	Tanya Mailhot	4	2019	0.00
7	Robert Goldberg	4	2019	0.00
8	Andreas Goette	4	2013	0.00
9	Deirdre A. Lane	4	2009	0.00
10	Gregory Y. H. Lip	4	2007	0.00

Two or more authors who were cited by another or more papers at the same time constitute a co-cited relationship, and the two or more authors are co-cited authors. In this study, there are 10 co-cited authors who had been cited more than or equal to 22 times among 408 co-cited authors (Table 2). Among the top 10 co-cited authors, the centrality of Zigmond AS, Dorian P, and Frasure-Smith N was more than 0.1.

**Table 2:** Top 10 co-cited authors on atrial fibrillation and depression.

**Rank**	**Co-cited author**	**Citation**	**Centrality**
1	Thrall G	57	0.02
2	Zigmond AS	34	0.19
3	Mccabe PJ	33	0.01
4	Dorian P	33	0.16
5	Lane DA	31	0.00
6	Lange HW	29	0.08
7	Kirchhof P	26	0.00
8	Frasure-Smith N	24	0.10
9	Camm AJ	24	0.00
10	Ware JE	22	0.04

### Distribution of Countries/Territories and Institutions

**Figure 4:**
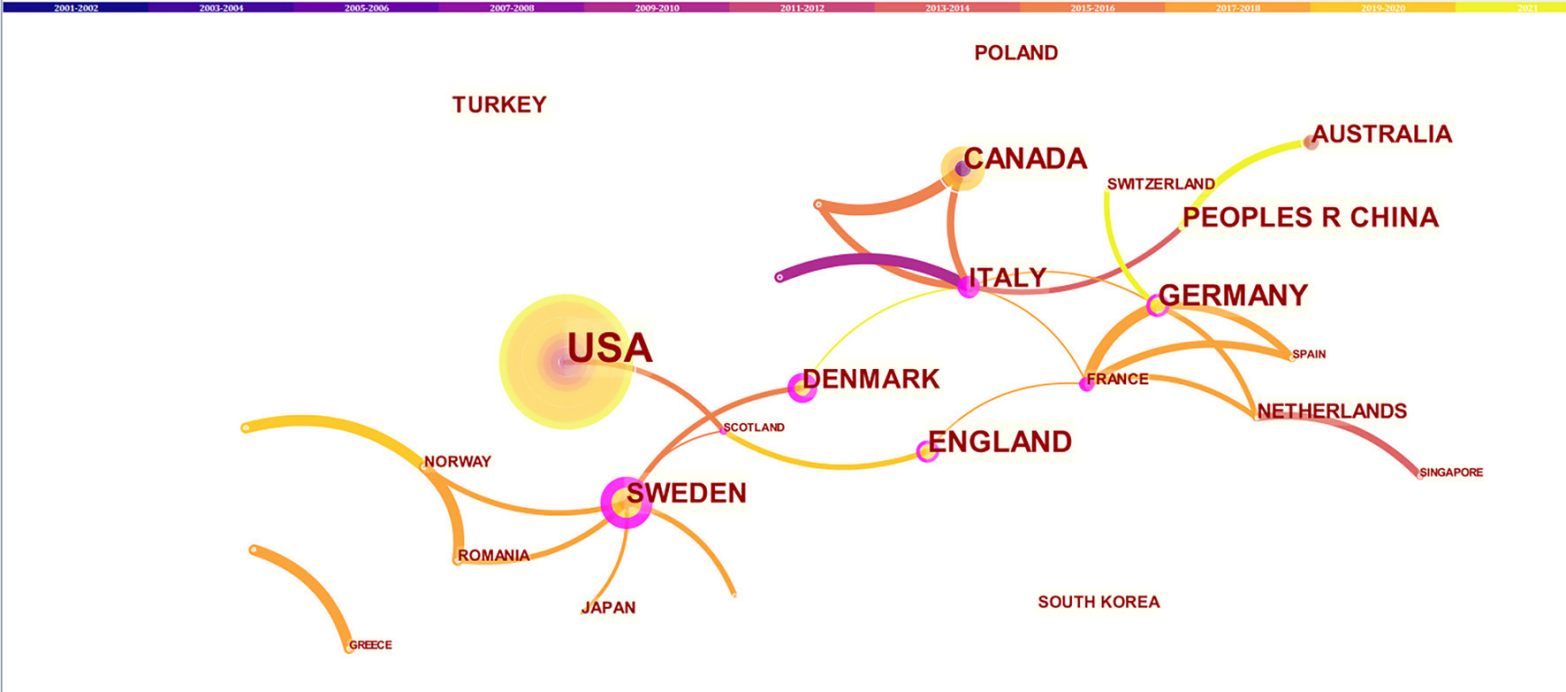
The network of countries/territories engaged in the research about atrial fibrillation and depression. In the networks, the larger the node was, the more contribution the country/territory had made to that field. The nodes with higher centrality (>0.1) are highlighted with purple rings. The color of the line represents the time of first co-occurrence. The thicker the line is, the greater the connection strength is (calculation method based on cosine).

203 Institutions From 30 Countries/Territories had co-Authored 159 Publications on AF and Depression. As shown in Figure 4 (Nodes = 30, Links = 51), the USA had the highest number of publications (54), followed by Canada (15), Germany (15), England (15), China (13), Sweden (13), Italy (12) and Denmark (11). Researches in the field had sprung up in the USA since 2002, while researches in this field had only emerged in China since 2012. Among them, the countries whose centrality is 0.1 were Italy (0.60), Sweden (0.47), Denmark (0.34), Germany (0.18), England (0.13), and China (0.10).

**Figure 5:**
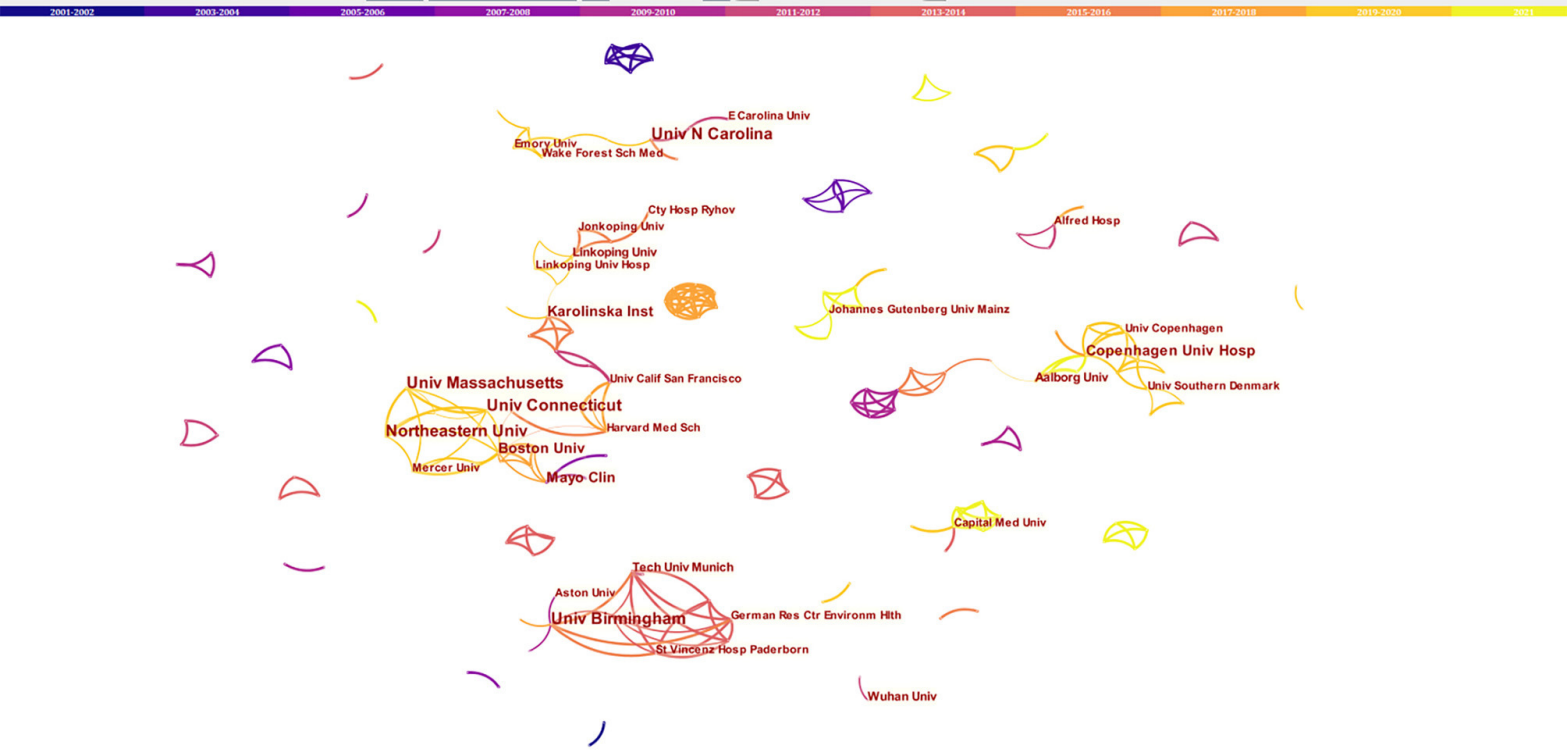
The network of institutions engaged in the research about atrial fibrillation and depression. In the networks, the larger the node was, the more contribution the institution had made to that field. The color of the line represents the time of first co-occurrence. The thicker the line is, the greater the connection strength is (calculation method based on cosine).

The network of co-occurrence institutions was shown in Figure 5 (Nodes = 203, Links = 270). The top 5 institutions with a large number of papers were Northeastern University, University of Birmingham, University of North Carolina, University of Connecticut, and University of Massachusetts, of which 4 were in the USA. The centrality of the above-mentioned institutions was all <0.01, and the cooperation among different institutions was less.

### Analysis of Co-cited References

Two or more references which were cited by another or more papers at the same time constitute a co-cited relationship, and the two or more references are co-cited references. The analysis of publications with high co-citation frequency can help to understand the basis of subject research. The 159 studies on AF and depression in recent 20 years involved 416 co-cited references, of which 5 studies were co-cited more than or equal to 13 times with centrality more than 0.10 (Table 3).

**Table 3:** Top 5 co-cited references on atrial fibrillation and depression.

**Rank**	**Author**	**Year**	**References**	**Citation**	**Centrality**
1	Thrall G et al.	2007	Depression, anxiety, and quality of life in patients with atrial fibrillation	23	0.35
2	Gehi AK et al.	2012	Psychopathology and Symptoms of Atrial Fibrillation: Implications for Therapy	17	0.19
3	Coulthard K et al.	2013	A feasibility study of expert patient and community mental health team led bipolar psychoeducation groups: implementing an evidence based practice	16	0.10
4	Lange HW et al.	2007	Depressive symptoms predict recurrence of atrial fibrillation after cardioversion	16	0.20
5	Dabrowski R et al.	2010	Quality of life and depression in patients with different patterns of atrial fibrillation	13	0.25

### Analysis of Keywords

**Figure 6:**
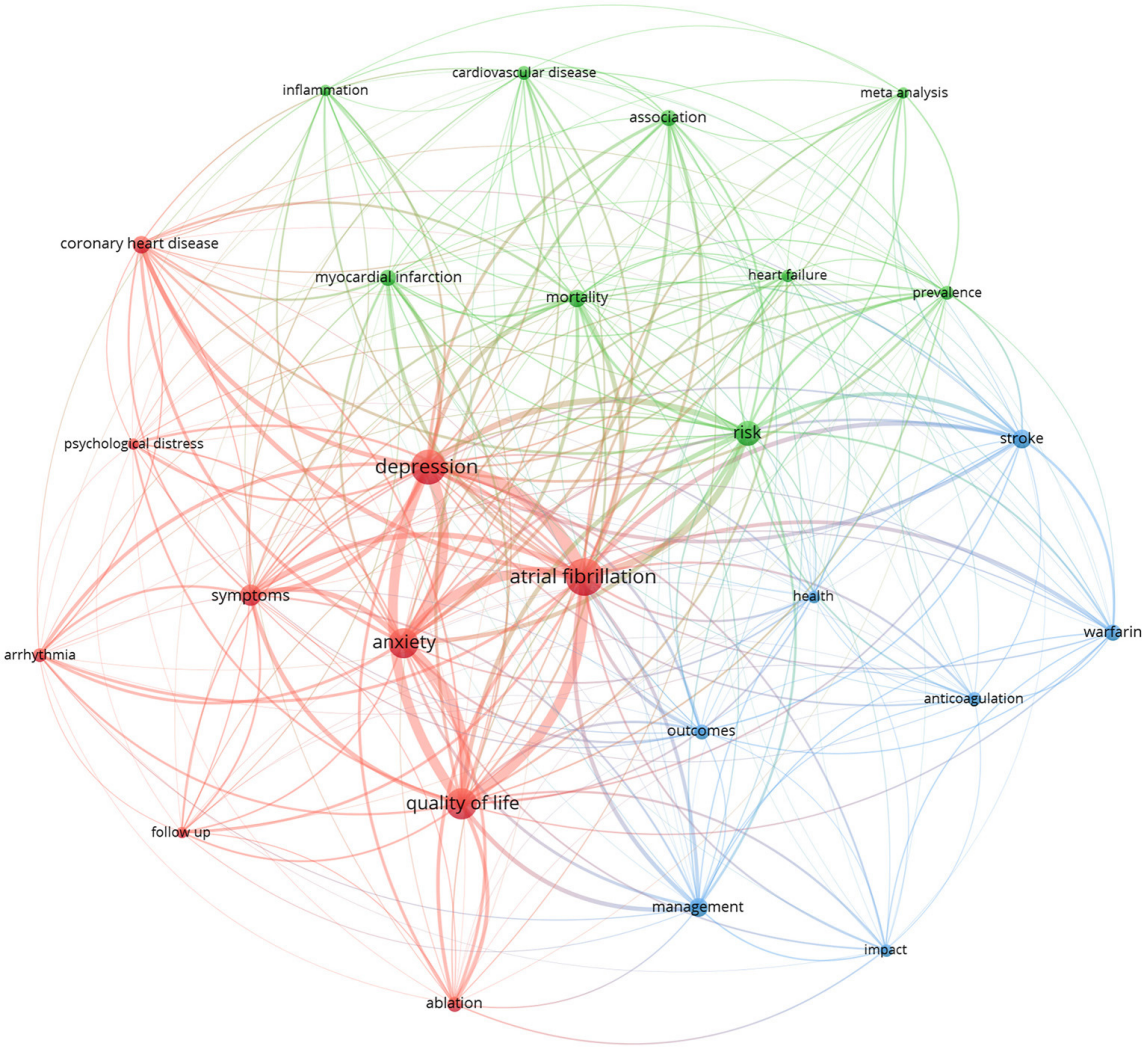
The network visualization of keywords. The size of each circle represents the weight of a keyword. The distance between two circles indicates the relatedness between two circles. The stronger the relatedness, the shorter the distance. The color of the circles represents the respective cluster class.

The key word is the core of an article. The analysis of the key words of the paper can peep into the theme of the article. We can find the hotspots in specific research fields by analysis of keywords. The top 20 keywords in occurrence frequency were shown in Table 4. The network visualization of keywords was conducted by VOSviewer and shown in Figure 6. The clusters of red, blue, and green, indicating three research directions. The keywords of green cluster were risk, prevalence, mortality, heart failure, association, myocardial infarction, cardiovascular disease, inflammation, and meta- analysis. The keywords of red cluster were composed of atrial fibrillation, depression, anxiety, symptoms, quality of life, ablation, symptoms, coronary heart disease, psychological distress, arrhythmia, follow up. While the keywords of blue cluster included outcomes, management, health, anticoagulation, impact, stroke, and warfarin.

**Table 4:** Top 20 keywords on atrial fibrillation and depression.

**Rank**	**Keywords**	**Count**	**Centrality**	**Rank**	**Keywords**	**Count**	**Centrality**
1	atrial fibrillation	104	0.03	11	myocardial infarction	23	0.11
2	depression	94	0.20	12	stroke	22	0.01
3	quality of life	72	0.01	13	ablation	21	0.06
4	anxiety	65	0.21	14	mortality	21	0.04
5	risk	48	0.01	15	outcome	21	0.03
6	symptom	31	0.00	16	association	20	0.42
7	anticoagulation	28	0.05	17	warfarin	18	0.10
8	management	27	0.14	18	arrhythmia	16	0.19
9	coronary heart disease	25	0.23	19	cardiovascular disease	16	0.07
10	epidemiology	23	0.04	20	heart failure	12	0.06

**Figure 7:**
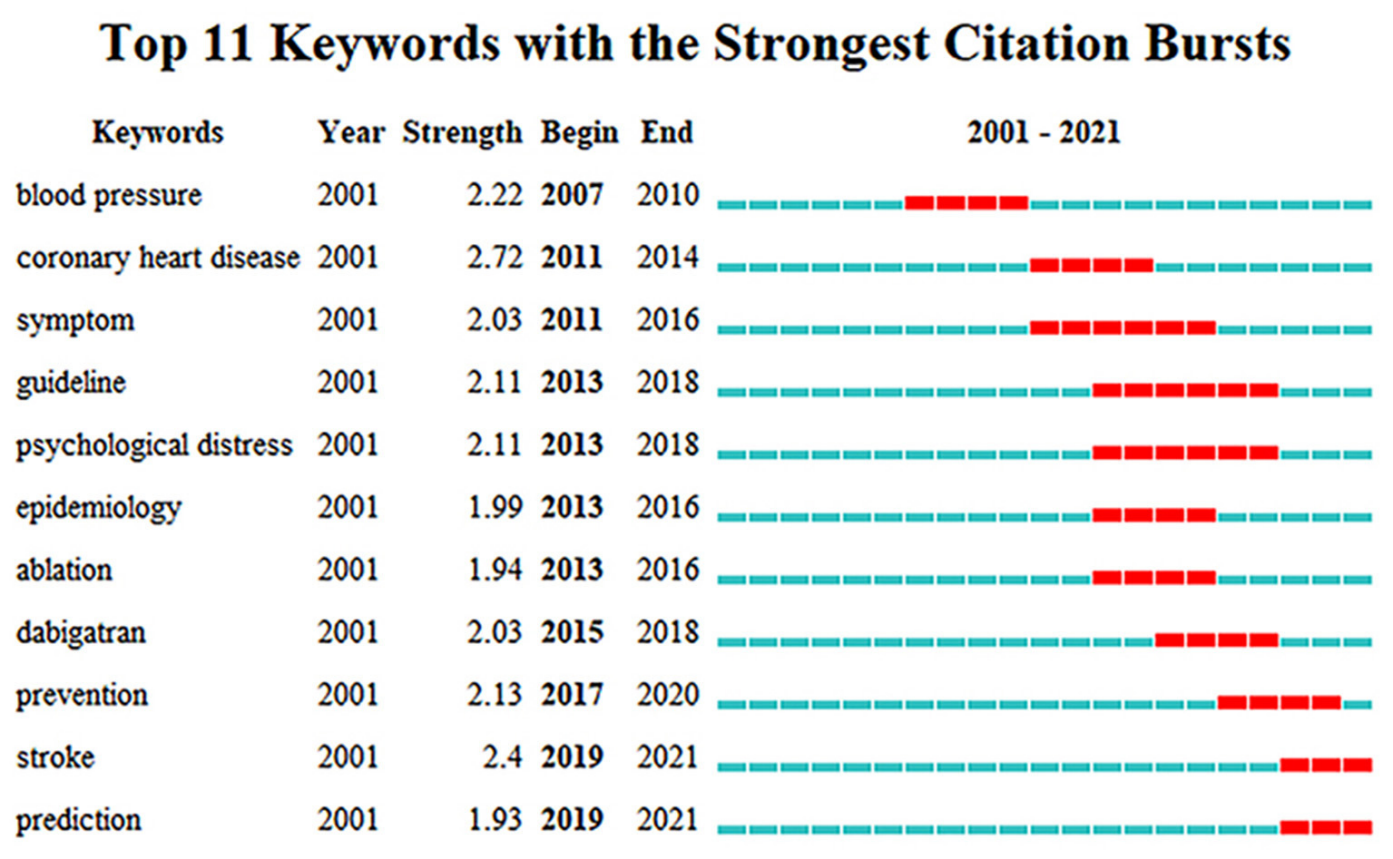
Top 11 keywords with the strongest citation bursts. Begin and End represent the beginning and end years of keyword emergence respectively. Strength indicates the intensity of the cited change. Each red or blue bar represents the time interval, and a single bar is equal to one year. The red bar especially represents citation burst.

Burst detection is a class of algorithms to identify changes of a variable over a period of time with reference to others in the same population, providing evidence that a particular publication is associated with a surge of citations, and the keyword citation burst was supported by CiteSpace based on Kleinberg's algorithm (18). The strength-value is an index measuring the strength of citation burst. And the higher the value, the stronger the burst. The minimum duration was set as 2 years, γ = 0.6. A total of 11 keywords with the strongest citation bursts were detected and sorted by the beginning years of citation burst (Figure 7). Strength indicates the intensity of the cited change. The strength of the 11 keywords was 1.93–2.72, and the duration of the bursts was 3–6 years. After 2019, stroke and prediction are cited frequently.

## Discussion

### General Information

Overall, the number of publications between 2001 and 2021 was small, which demonstrated that the studies during this period were lacking, suggesting that research on AF and depression is an emerging field that is not in-depth. As shown in Figure 2, the maximum number of annual publications was 11 in 2001–2017, indicating that researchers had just realized that AF is related to depression. In 2018, the number of articles published increased sharply, reaching 22 articles, and the annual number of articles remained at about 20 from then. The sharply growth period being from 2018 to 2021, the number of publications totalled 75, accounting for 47.2% of all publications. Is the increase in publications regarding AF and depression associated with a general increase in publications in the field of AF or depression? According to the bibliometric studies, we can learn about that the annual publication of AF or depression had increased steadily year by year from 2015 to 2018, but there is no rapid growth period (19, 20). From this point of view, the sharp increase in the number of annual publication on AF and depression in recent years may not be entirely related to the increase in the publication of AF or depression, but it may be because scientists pay more attention to the association between on AF and depression. Over the past years, more studies have focused on the impact of depression on the occurrence, development, and therapy of AF, suggesting the research on AF and depression is likely to become a hot topic and research direction in the future.

The cooperation network analysis of authors can provide information on influential research groups and potential collaborators and is helpful to establish collaborations for researchers (21). As shown in Figure 3, lots of authors were involved in this field. However, the cooperation among authors needs to be strengthened. The top 10 authors on AF and depression (Table 1) were the potential collaborators. Among the top 10 co-cited authors, the centrality of Zigmond AS, Dorian P, and Frasure-Smith N was more than 0.1, indicating that they are influential authors in the field and reading their publications was beneficial to understanding the knowledge structure in the field. Through visual analysis of the distribution of countries/territories and institutions, we can learn about that the USA was the leading where the study on AF and depression was occurring. There were more productive authors in the USA, and 7 of the top 10 productive authors worked in the USA. In addition, American authors have studied AF and depression for the longest time, starting in 2002. As a result, the USA has the most publications, and research on AF and depression may be more mature than in other countries. But the centrality of USA was <0.1, indicating a lack of cooperation with other countries/territories. As shown in Figure 5, most of the institutions with prolific publications were in the USA. The scientific research cooperation among institutions was greatly influenced by the region. Therefore, it is strongly suggested that research institutions and researchers from different countries actively cooperate and communicate rather than retain academic barriers to promote the research and development of AF.

The top 5 co-cited references were mainly related to the following aspects attracting researchers: ➀ Correlation between psychosocial problems (depression, anxiety, etc.) and adverse outcomes in patients with AF (2, 5, 11, 22); ➁ The acceptability and feasibility of a group psychoeducation program delivered by community mental health teams (CMHTs) and peer specialist facilitators (23). These literatures laid the foundation for the study of AF and depression. As researchers had realized that the depression was closely related to the occurrence and development of AF, which increased the complexity of management and the risk of adverse outcomes in patients with AF, the importance of psychoeducation began to pay attention. Nevertheless, the treatment of patients with AF and depression is still a challenge.

### The Hotspots and Frontiers

Keyword analysis can help us understand the hotspots and frontiers in a particular field. It can be seen from Table 4 that the keywords with high occurrence frequency were atrial fibrillation, depression, quality of life, anxiety, risk, symptom, anticoagulation, management, coronary heart disease, epidemiology, myocardial infarction, stroke, ablation, mortality, outcome, association, et al. Cluster analysis was conducted based on keywords, and three clusters were formed finally. Keywords are the research themes and core contents of the literature. There were 3 clusters in network visualization of keywords (Figure 6), which reflected the research hotspots in the past 20 years. By comprehensive keyword analysis, we can learn that researchers mainly focused on the following three aspects.

The first was the effect of depression on prevalence and mortality in AF. In the past 20 years, many studies had explored the effects of depression on the incidence and mortality in AF, involving the effect of depression on the incidence of cardiovascular disease (including AF, myocardial infarction and heart failure, et al.), and the association between depression and the increased risk of mortality in AF. Inflammation may be one of the mechanism of the relationship between depression and AF. Meta-analysis was an important research method to evaluate the association between AF and depression. Therefore, we speculated that the keywords of green cluster (Figure 6) are related to the theme of “Effect of depression on prevalence and mortality of AF.” The studies found that the incidence of depression was significantly higher in patients with AF than that in patients without AF (2, 24, 25), but it is unclear whether depression contributes to AF or vice versa. Most scholars believed that the high burden of AF symptoms and decreased quality of life are the main causes of depression (2). On the other hand, a growing number of studies suggested that depression can cause AF. The large multicenter prospective cohort study conducted by Garg, P. K. et al. (involving 6,644 participants detected over a median follow-up of nearly 13 years) suggested that depression was associated with an increased risk of AF (10). Another study of 37,402 adult residents with an average of 8.1 years of follow-up found that mild to moderate depression symptoms were associated with an increased risk of AF (26). Similar to the mechanism of depression affecting other disease (heart failure, myocardial infarction, cancer, et al.), the mechanism of depression affecting AF may be related to inflammation, oxidative stress, autonomic nerve function, hypothalamic-pituitary-adrenal axis imbalance, and so on (27–29). Several studies had found that elevated depression symptoms predicted cardiovascular mortality and all-cause mortality in patients with AF (30). The association between AF and depression has been widely recognized in the academic community, but the causal relationship needs to be further explored in higher quality prospective cohort studies. In addition, Further study into whether improving depressive symptoms reduces morbidity and mortality in patients with AF is important too (30).

The second was depression on ablation in AF. Symptom relief and quality of life improvement are important goals of health management for patients with AF. Several studies have shown that depression is significantly associated with increased symptom burden and decreased quality of life in patients with AF, which increases the readmission rate (22, 26, 31–34). Catheter ablation is an important means to reduce the symptom burden and improve the quality of life of patients with AF (35). Through the follow-up of patients with AF after ablation, we can judge the curative effect of ablation and the effect of psychological distress (including depression and anxiety) on it. After excluding the key word “coronary heart disease,” which was related to common complications of AF, other keywords of the red cluster (involving atrial fibrillation, depression, anxiety, symptoms, quality of life, ablation, symptoms, arrhythmia, and follow up) are all related to the theme “Depression on ablation in AF.” Catheter ablation is an important non-drug method of cardioversion, achieving restoration and maintenance of sinus rhythm. Increasing numbers of patients receive AF ablation nowadays. However, a meta-analysis of seven cohort studies with 1,070 AF patients who underwent catheter ablation by circumferential pulmonary vein isolation indicated depression was an independent risk factor of AF recurrence after catheter ablation (36). The underlying mechanism of depression affecting the outcomes of AF after ablation is still unclear. It has been proposed that adherence to medication in patients with depression was poor (37). The mechanism may be related to drug compliance of patients with AF after ablation, and it may also be related to depression-related inflammation, oxidative stress, autonomic nerve function, hypothalamic-pituitary-adrenal axis imbalance, and so on. In view of the great and complex influence of depression on ablation, treatment of AF and psychological comorbidities may be beneficial. The exact mechanism of depression affecting the efficacy of ablation in AF patients remains to be clarified, which provide the basis for finding effective measures to reduce the recurrence rate after ablation, reduce symptom burden and improve quality of life.

The third was the impact of depression on anticoagulation treatment in AF. The keywords of blue cluster included outcomes, management, health, anticoagulation, impact, stroke, and warfarin. AF is associated with an increased risk of thrombotic events and leads to a nearly 5-fold increase in the risk of stroke (38, 39). Anticoagulant therapy is an important part of the management of patients with atrial fibrillation to prevent stroke and other adverse outcomes. Oral anticoagulants, including direct oral anticoagulants (DOACs) and warfarin), are the cornerstone of stroke prevention in high-risk patients with AF (40). AF patients who are at high risk for stroke should receive anticoagulation therapy unless contraindicated. Depression affecting anticoagulant therapy in patients with AF is a serious threat to their health. Thus, we estimated that keywords of blue cluster were all relevant to the topic “Impact of depression on anticoagulation treatment in AF.” The retrospective cohort study including 31 951 veterans with AF and case-control study (157 cases and 329 control subjects) had both shown that depression can increase the risk of bleeding, which was the most common and serious side effect of anticoagulation therapy (41, 42). A prospective cohort study (thrombEVAL study) including *n* = 1,558 patients who received oral anticoagulation (OAC) therapy with vitamin K antagonists revealed that depressive symptoms might be a risk factor for clinically relevant bleeding incidents too (43). Possible underlying mechanisms for the increased bleeding risk might be poor adherence to the treatment regimens in depression patients (44). Whether the effects of antidepressants increase the risk of bleeding is still controversial (43, 45). Whether there are special pathological changes in patients with AF accompanied by depression that increase the risk of bleeding remains to be further studied *in vivo* and *in vitro*. It is necessary to eliminate influencing factors that affect the quality of anticoagulant therapy and so improve the chance of avoiding death or dependence after stroke.

The above three aspects are the hot spots in the field of AF and depression in the past 20 years, but there are still some problems that need further in-depth study to solve. Interestingly, the main hotspots varied with different periods as shown in Figure 7. Before 2007, the number of annual publications was very small, and in this study we did not detect citation burst keywords. Since 2007, the top 11 keywords with the strongest citation bursts was detected. Blood pressure, coronary heart disease and symptom were the top three keywords with citation burst in chronological order, suggesting that the influencing factors in AF (symptom burden, incidence of complications, etc.) were still being explored, and hypertension and coronary heart disease were the factors that researchers pay more attention to than depression. With a growing number of studies had found that depression may be related to AF. The keywords psychological stress, epidemiology and ablation were detected in 2013, suggesting that researchers began to pay attention to the prevalence of psychological stress including depression among patients with AF and its influence on ablation in AF. The key words dabigatran, prevention, stroke and prediction, all related to anticoagulation therapy, have burst one after another since 2015. Stroke is the main complication of patients with AF, which seriously endangers the life and health of patients. In 2015, A case report pointed out that depressive symptoms associated with dabigatran (a DOAC) in 2015 (46), and then researchers began to pay more attention to the association between depression and anticoagulants. It was found that depression was related to the increased risk of bleeding on anticoagulants. Prevention of stroke in patients with AF is the primary task of AF treatment, but the mechanism of depression predicting the risk of stroke in patients with AF has not been clarified, and how to eliminate the influence of depression on anticoagulants remains to be solved. It is worth noting that the citation burst period of keywords stoke and prediction continues to 2021, indicating that the research on stroke prevention (including anticoagulant treatment) may still be the focus of research in the next few years.

Except for ablation and anticoagulation treatment related to the management of AF patients itself, no keywords referring treatment were found in keywords analysis (either high-frequency keyword clustering analysis or keywords with strongest citation burst analysis). Despite the research hotspots, which is mainly focus on the adverse effects of depression on patients with AF, limited studies have explored treatment strategies for patients with AF complicated with depression, and effective therapeutic intervention for that remains a challenge. Researchers are beginning to look at educational interventions (47, 48) and Yoga (49), but more data is needed on the their clinical benefit. It is still of potential value to develop drugs with new mechanisms. Experimental data from animal studies supported the idea that the Sigma-1 receptor agonist (SA4503) might be the promising pharmacological agent to treat depression-related AF by increasing conduction function, improving the expression of connexin 40 and 43, and reducing cardiac myocardial inflammation, but it has not been used in clinical practice (50, 51). Traditional Chinese medicine (TCM) has drawn great attention because of its relative safety and long history, which is an integral part of mainstream medicine in China (52). TCM has unique advantages in the treatment of patients with AF complicated with depression. However, at present, due to the lack of international cooperation and communication, there are few English publications related to its clinical application and mechanism, but mainly Chinese publications. It would be meaningful to evaluate whether standard of care for depression in patients for other disease (heart failure, coronary heart diseases, cancer, et al.) could be also used for patients with AF. The optimal treatment of AF combined with depression needs further exploration, especially the treatment that can improve the long-term prognosis.

### Strength and Limitation

To our knowledge, this is the first bibliometric analysis of AF and depression. The visual analysis provides channels for researchers to understand the research subjects, hotspots, and development trends in the field of depression and AF. But some limitations should be addressed. First, in order to meet the reference format requirements of CiteSpace, this study only retrieved literature from the WOSCC. Web of Science is an important data platform to obtain global academic information and one of the most authoritative scientific and technological literature retrieval tools, which can provide important research information in the field of science and technology, but it cannot cover the research contents of AF and depression. Second, several keywords based on software extraction may not be appropriate, and it's necessary to understand the meaning of keywords comprehensively in combination with the researcher's personal understanding, so there may be bias. Third, the literature information in 2021 has not been fully counted because of the limitation of extraction deadline. Finally, the uneven quality of the collected literature may reduce the credibility of the map analysis.

## Conclusion

Depression is closely related to the occurrence and development of AF, which increases the complexity of management and the risk of adverse outcomes in patients with AF. However, the current research is still in the initial stage, and there are lots of issues to be explored. It is imperative to increase international cooperation and carry out more in-depth research in this field. The effect of depression on prevalence and mortality in AF, depression on ablation in AF, and impact of depression on anticoagulation treatment in AF have been the focus of current research. Stroke prevention (including anticoagulant therapy) may still be the focus of research in the future. This suty can provide relevant researchers with research hotspots and frontiers in this field, provide ideas for finding new topics and directions, and promote the further development of research on AF and depression.

## Data Availability Statement

The original contributions presented in the study are included in the article/supplementary material, further inquiries can be directed to the corresponding authors.

## Author Contributions

JZ and XM conceived and supervised the research. YA designed this study, collected the data, performed the data analysis, and drafted the manuscript. YX collected the data. LY, DM, and AG rechecked the data. QX and SZ created the illustrations. TM and QP provided valuable suggestions for data analysis and figure design. All the authors reviewed and approved the submitted version of the article.

## Funding

This study was financially supported by the CACMS Innovation Fund (Grant No. CI2021A00915) and National Natural Science Foundation of China (Grant No. 81573817).

## Conflict of Interest

The authors declare that the research was conducted in the absence of any commercial or financial relationships that could be construed as a potential conflict of interest.

## Publisher's Note

All claims expressed in this article are solely those of the authors and do not necessarily represent those of their affiliated organizations, or those of the publisher, the editors and the reviewers. Any product that may be evaluated in this article, or claim that may be made by its manufacturer, is not guaranteed or endorsed by the publisher.
